# Supplementary material for: Investigating the volume and diversity of data needed for generalizable antibody–antigen ΔΔG prediction
Source: Nat Comput Sci. 2025 Jul 8;5(8):635–47. doi: 10.1038/s43588-025-00823-8 (PMC12374841; doi:10.1038/s43588-025-00823-8)
Supplement: Supplementary file 1 — Supplementary Figs. 1–10, Tables 1–6 and results. [file 43588_2025_823_MOESM1_ESM.pdf]

nature computational science

Supplementary information https://doi.org/10.1038/s43588-025-00823-8

Investigating the volume and diversity of data needed for generalizable antibody–antigen ΔΔ*G* prediction

In the format provided by the authors and unedited

Supplementary Information Contents

Supplementary Figures 2

Supplementary Figure 1. The distributions of the ΔΔG values of the base datasets used in this study. 2

Supplementary Figure 2. The Pearson's correlations of Graphinity on different train-validation-test cutoffs applied to the Experimental_ΔΔG_645 and Synthetic_FoldX_ΔΔG_942723 datasets. 3

Supplementary Figure 3. The performance of Graphinity and a tree-based model on the Experimental_ΔΔG_608 dataset. 3

Supplementary Figure 4. Correlations between synthetic and experimental ΔΔG values, applied to the Experimental_ΔΔG_608 dataset. 4

Supplementary Figure 5. The performance of Graphinity on the Synthetic_FoldX_ΔΔG_942723 dataset, with the train, validation, and test data split on the basis of affinity. 5

Supplementary Figure 6. The performance of Equiformer models developed with varying amounts of synthetic FoldX train plus validation data. 5

Supplementary Figure 7. The performance of Graphinity on synthetic data generated using Flex ddG and FoldX. 6

Supplementary Figure 8. Synthetic FoldX ΔΔG values separated by amino acid substitution. 6

Supplementary Figure 9. Graphinity performance on amino acid substitutions in the synthetic FoldX dataset. 7

Supplementary Figure 10. Graphinity scoring of 36,391 Trastuzumab CDRH3 variants. 7

Supplementary Tables 8

Supplementary Table 1. Descriptions of the experimental and synthetic ΔΔG datasets used in this study. 8

Supplementary Table 2. The performance of ΔΔG prediction methods trained on experimental antibody-antigen ΔΔG datasets. 9

Supplementary Table 3. Synthetic ΔΔG dataset generation runtimes. 9

Supplementary Table 4. The performance of different models on the Synthetic_FoldX_ΔΔG_942723 dataset. 9

Supplementary Table 5. Pharmacophore counts for each amino acid, as used in the tree-based model featurization. 9

Supplementary Table 6. Structural changes resulting from modeling of mutations using FoldX and Flex ddG. 9

Supplementary Results 11

References 12

Supplementary Figures

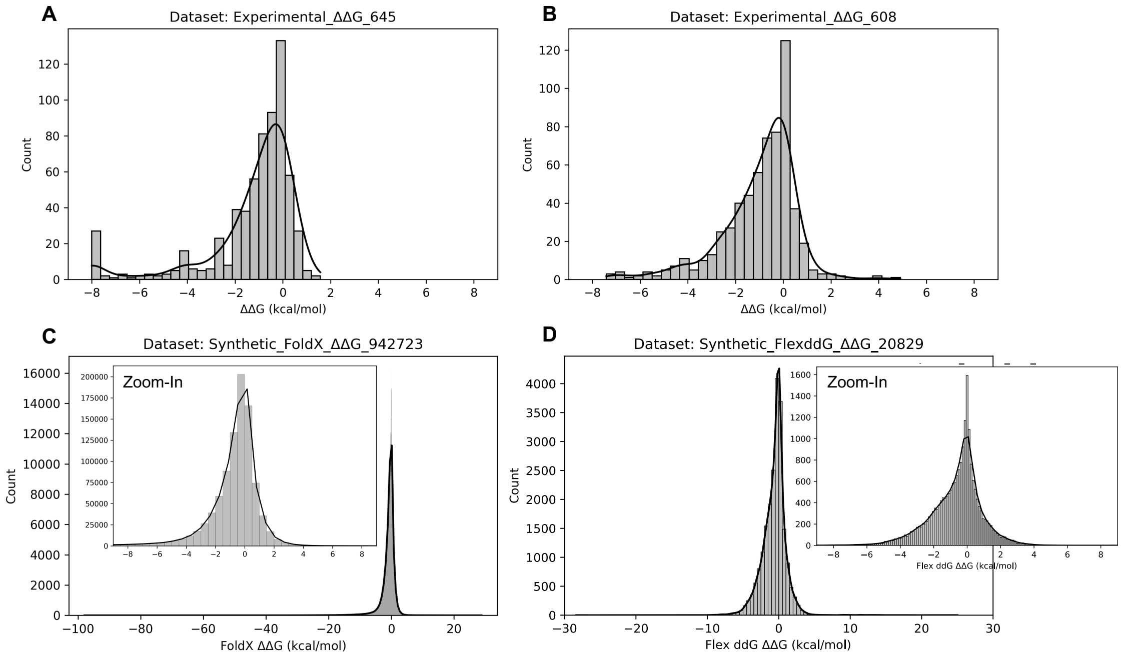

Supplementary Figure 1: The distributions of the ΔΔG values of the base datasets used in this study: (A) Experimental_ΔΔG_645, (B) Experimental_ΔΔG_608, (C) Synthetic_FoldX_ΔΔG_942723, (D) Synthetic_FlexddG_ΔΔG_20829. The solid lines are kernel density estimates.

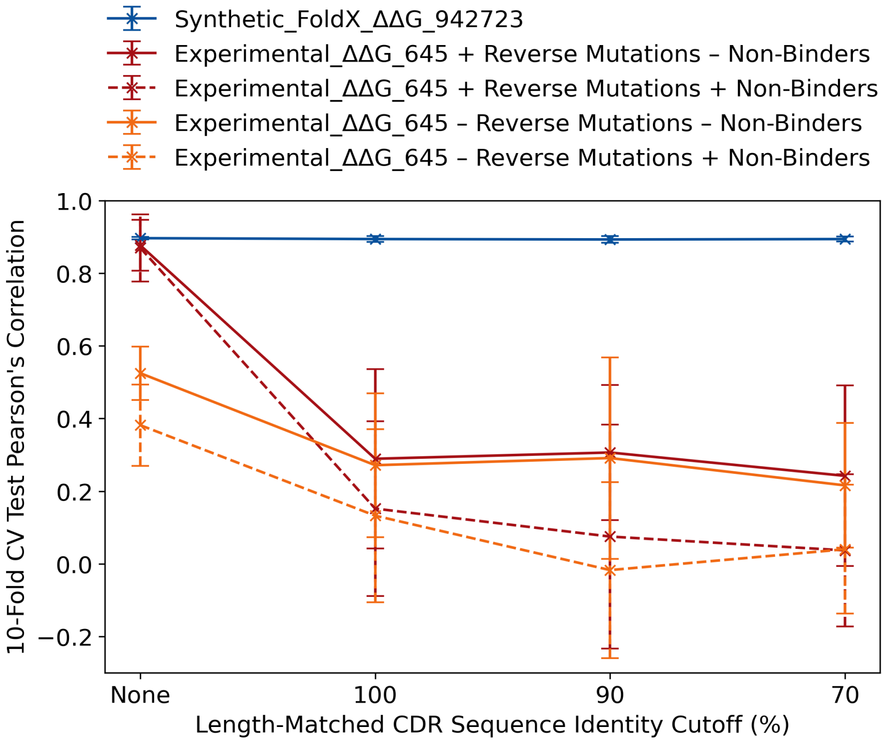

Supplementary Figure 2: The Pearson's correlations of Graphinity on different train-validation-test cutoffs applied to the Experimental_ΔΔG_645 dataset (red, orange) and Synthetic_FoldX_ΔΔG_942723 dataset (blue). This is Figure 2B including error bars, which represent the standard deviation in Pearson's correlation across the 10 folds of 10-fold cross-validation (CV).

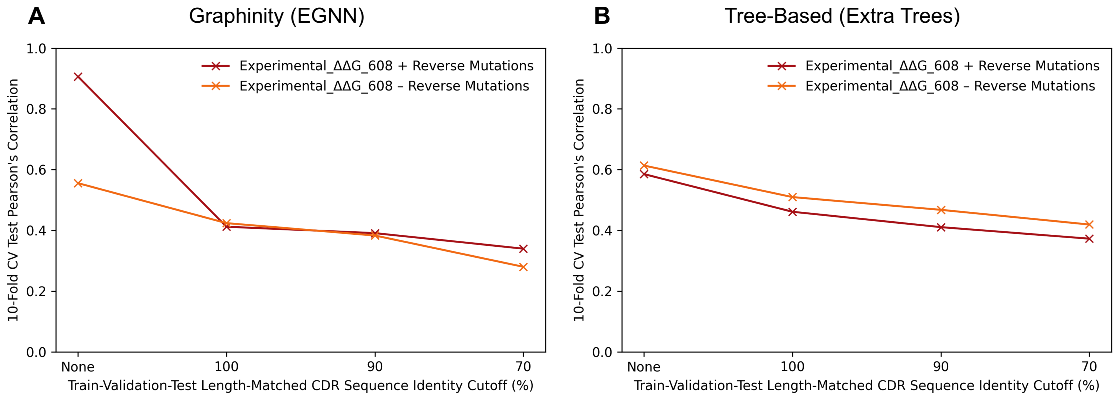

Supplementary Figure 3: The performance of (A) Graphinity (EGNN architecture) and (B) a tree-based (Extra Trees) model on the Experimental_ΔΔG_608 dataset, with and without reverse mutations, at different length-matched CDR sequence identity cutoffs.

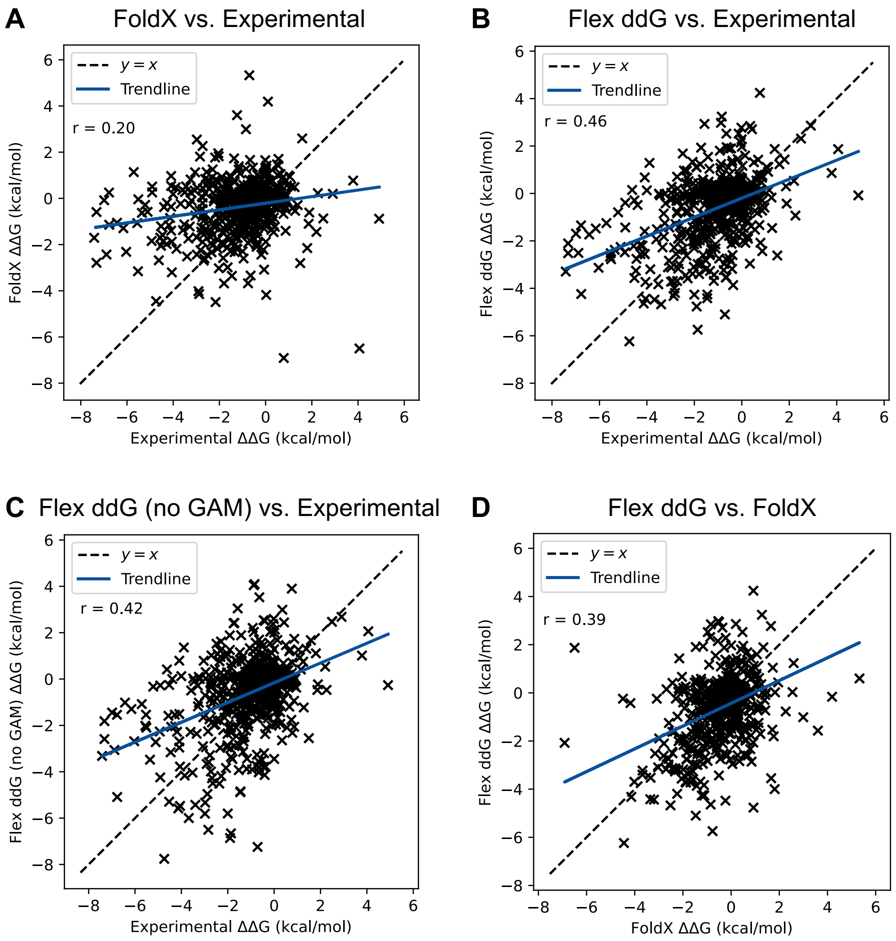

Supplementary Figure 4: Correlations between synthetic and experimental $\Delta\Delta G$ values, applied to the Experimental_$\Delta\Delta G$_608 dataset. (A) FoldX vs. experimental values. (B) Flex ddG (Talaris 2014 forcefield with GAM reweighting) vs. experimental values. (C) Flex ddG (Talaris 2014 forcefield without GAM reweighting) vs. experimental values. (D) Flex ddG (Talaris 2014 forcefield with GAM reweighting) vs. FoldX values. The Pearson's correlation (r) values are indicated. The trendlines, shown in blue, are least squares polynomial fits.

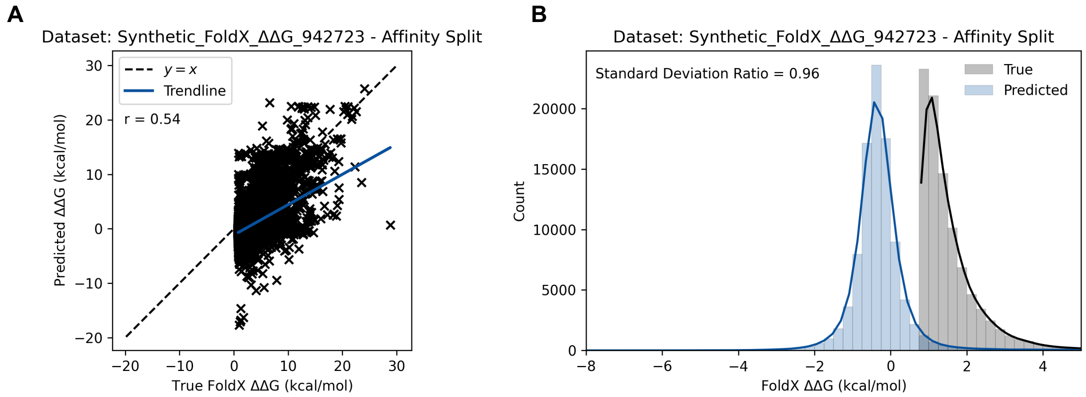

Supplementary Figure 5: The performance of Graphinity on the Synthetic_FoldX_ΔΔG_942723 dataset, with the train, validation, and test data split on the basis of affinity (bottom 80%, next 10%, top 10%, respectively). (A) Correlation between and (B) distribution of true and predicted values on the test set (top 10% of affinity values in the full dataset). The Pearson's correlation (r) value is shown in (A).

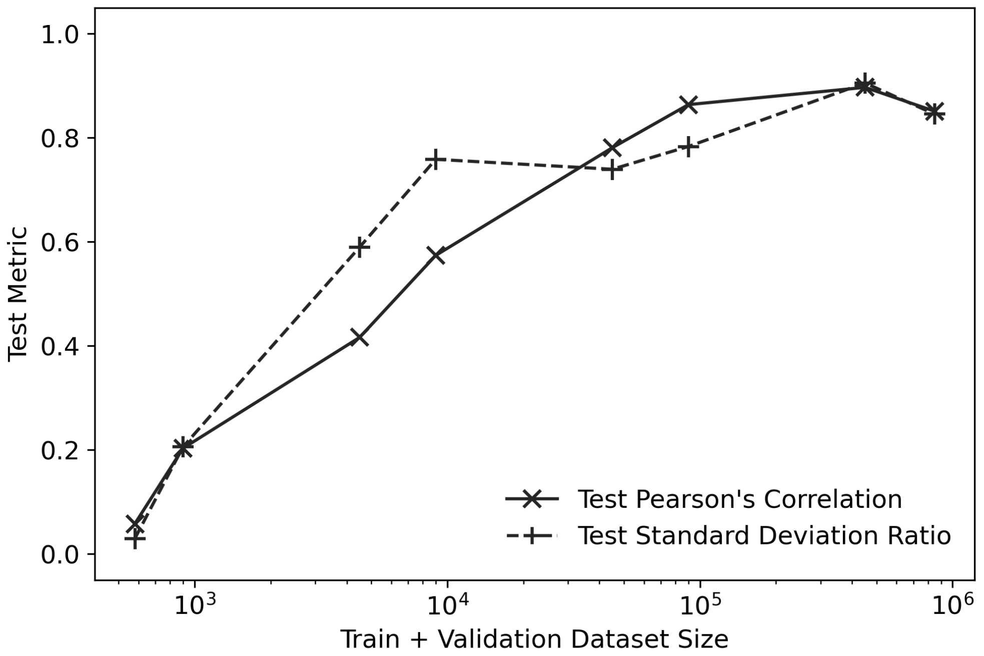

Supplementary Figure 6: The performance of Equiformer models developed with varying amounts of synthetic FoldX train plus validation data. Datasets used: Synthetic_FoldX_ΔΔG_580-450000 (Supplementary Table 1). All models were evaluated on the same test set, consisting of 94,126 mutations (one fold, held-out test set; 90% length-matched CDR sequence identity cutoff).

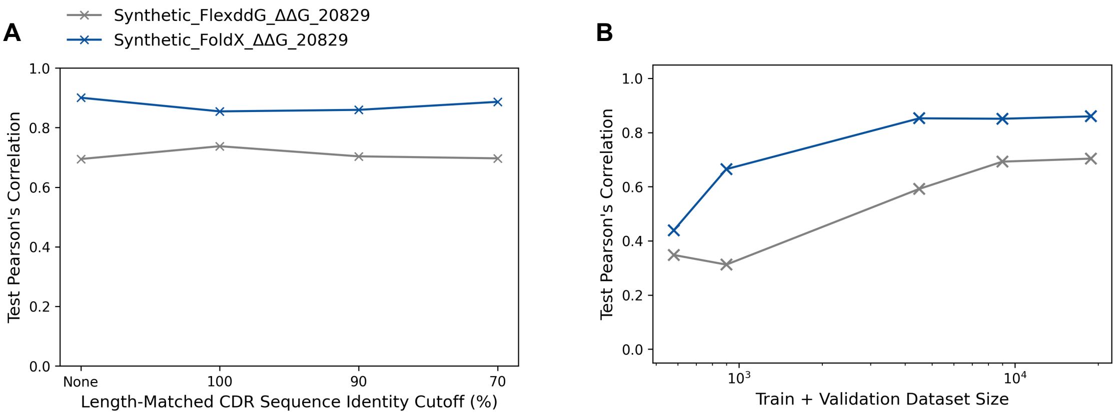

Supplementary Figure 7: The performance of Graphinity on synthetic data generated using Flex ddG and FoldX. (A) Test Pearson's correlations on the Synthetic_FlexddG_ΔΔG_20829 dataset and a subset of the Synthetic_FoldX_ΔΔG_942723 dataset comprised of the identical mutations, at different train-validation-test sequence identity cutoffs. (B) Test Pearson's correlations on varying amounts of train plus validation data from the Synthetic_FlexddG_ΔΔG_20829 dataset and subsets of the Synthetic_FoldX_ΔΔG_942723 dataset comprised of the identical mutations.

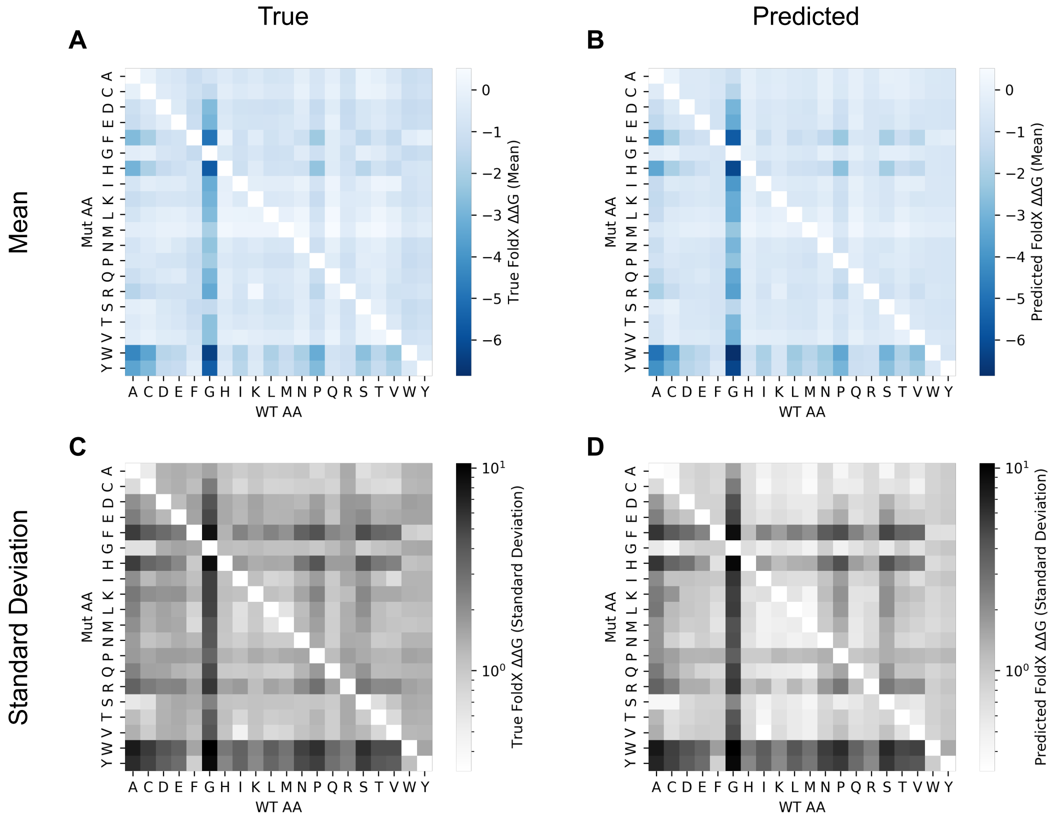

Supplementary Figure 8: (A,C) ΔΔG values from the Synthetic_FoldX_ΔΔG_942723 dataset, separated by amino acid substitution. (B,D) ΔΔG values predicted by Graphinity applied to the Synthetic_FoldX_ΔΔG_942723 dataset (10-fold cross-validation, 90% length-matched CDR sequence identity cutoff), separated by amino acid substitution. Top row: mean, bottom row: standard deviation. WT: wild-type, Mut: mutant.

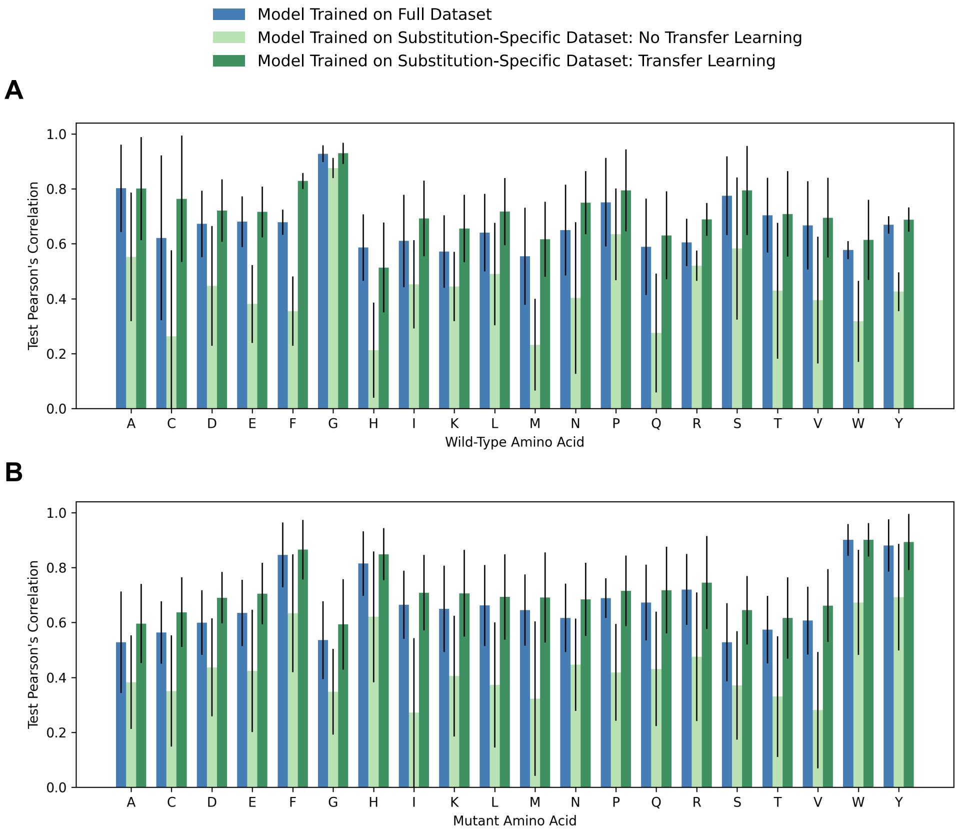

Supplementary Figure 9: Graphinity performance on amino acid substitutions when trained on the full dataset (blue; Synthetic_FoldX_ΔΔG_942723), substitution-specific dataset (light green), and substitution-specific dataset with weights initialized from the model trained on the full dataset (dark green). The results were grouped and averaged by (A) wild-type amino acid and (B) mutant amino acid. The error bars represent the standard deviation. For more details, see Supplementary Results ('Performance by amino acid substitution')

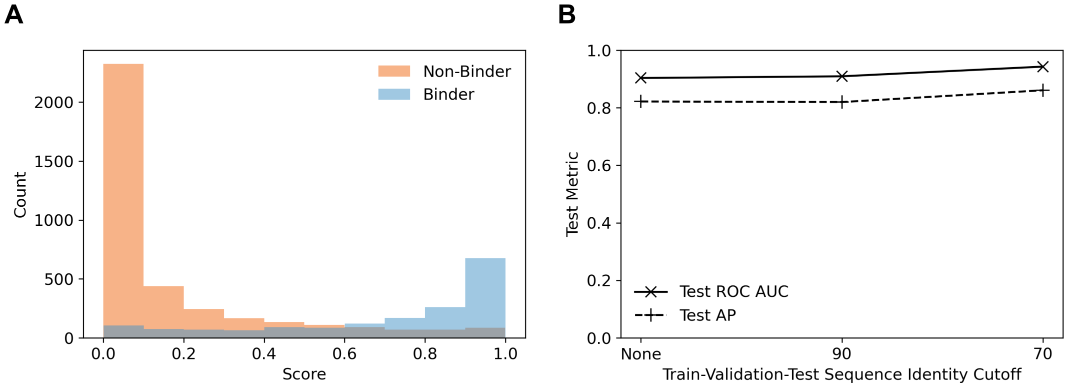

Supplementary Figure 10: (A) Graphinity scoring of 36,391 Trastuzumab CDRH3 variants [1] (randomly split data). (B) Model performance with clonotype and sequence identity cutoffs imposed between the train, validation and test datasets. In cases where a sequence identity cutoff (value shown on the x-axis) was applied, the data was also separated by clonotype (V- and J-gene assignments).

Supplementary Tables

Supplementary Table 1: Descriptions of the experimental and synthetic ΔΔG datasets used in this study. Ab: antibody, Ag: antigen, AA: amino acid. For definitions of inner and outer shell see Methods ('Varying synthetic dataset diversity').

Dataset Name	Experimental/ Synthetic	Description		Train-Val-Test Split (CDR Sequence Identity Cutoff)	Number of Mutations	Number of Complexes	Number of AA Substitution Types	Mutation Distribution (# of muts. in Ab Inner, Ab Outer, Ag Inner, Ag Outer)
Experimental_ΔΔG_645 – Reverse Mutations + Non-Binders	Experimental	Dataset of single-point mutations from AB-Bind (Sirin et al., 2016)	– reverse mutations, + non-binders	None (Random), 100%, 90%, 70%	645	24 (plus 5 homology models)	141	185, 148, 172, 140
Experimental_ΔΔG_645 – Reverse Mutations – Non-Binders			– reverse mutations, – non-binders		618		136	170, 136, 172, 140
Experimental_ΔΔG_645 + Reverse Mutations + Non-Binders			+ reverse mutations, +non-binders		1,290		224	370, 296, 344, 280
Experimental_ΔΔG_645 + Reverse Mutations – Non-Binders			+ reverse mutations, – non-binders		1,236		216	340, 272, 344, 280
Experimental_ΔΔG_608		Dataset of single-point mutations filtered from SKEMPI 2.0 (Jankauskaite et al., 2019)	– reverse mutations		608	44	163	232, 138, 151, 87
Experimental_ΔΔG_608 + Reverse Mutations			+ reverse mutations		1,216		232	464, 276, 302, 174
Synthetic_FoldX_ΔΔG_ 942723	Synthetic (FoldX)	Synthetic single-point mutation ΔΔG data generated using FoldX		None (Random), 100%, 90%, 70%, 70% + 70% Ag seq. identity cutoff	942,723	1,471	380	326,990, 155,439, 328,130, 132,164
Synthetic_FoldX_ΔΔG_ 942723_shuffled		Synthetic_FoldX_ΔΔG_942723 with a percentage of ΔΔG labels shuffled (i.e., incorrect)		90%				
Synthetic_FoldX_ΔΔG_ 942723_gaussian_noise		Synthetic_FoldX_ΔΔG_942723 with random noise sampled from Gaussian distributions with varying scales added						
Synthetic_FoldX_ΔΔG_580		Train + validation datasets of varying sizes randomly sampled from the respective Synthetic_FoldX_ΔΔG_942723 datasets			580	462	269	200, 87, 212, 81
Synthetic_FoldX_ΔΔG_900					900	645	313	296, 137, 331, 136
Synthetic_FoldX_ΔΔG_4500					4,500	1,264	377	1,519, 711, 1,602, 668
Synthetic_FoldX_ΔΔG_9000					9,000	1,316	380	3,052, 1,468, 3,180, 1,300
Synthetic_FoldX_ΔΔG_45000					45,000	1,324	380	15,522, 7,443, 15,843, 6,192
Synthetic_FoldX_ΔΔG_90000					90,000	1,324	380	31,337, 14,800, 31,387, 12,476
Synthetic_FoldX_ΔΔG_450000					450,000	1,324	380	156,449, 74,016, 156,945, 62,590
Synthetic_FoldX_ΔΔG_848597					848,597	1,324	380	294,614, 139,802, 296,305, 117,876
Synthetic_FoldX_ΔΔG_94126		Test dataset to which models trained on the train + validation datasets of varying sizes were applied			94,126	147	380	32,376, 15,637, 31,825, 14,288
Synthetic_FoldX_ΔΔG_ 100000_sequence_min		Datasets of 90,000 mutations sampled from Synthetic_FoldX_ ΔΔG_942723 to: [NB train and validation datasets only, which are 80,000 and 10,000 mutations respectively]	minimize antibody CDR sequence diversity		Train: 80,000; Val: 10,000	86 (Train + Val)	380	34,143, 10,678, 32,785, 12,394
Synthetic_FoldX_ΔΔG_ 100000_sequence_max			maximize antibody CDR sequence diversity			1,324 (Train + Val)	380	30,744, 15,420, 31,172, 12,664
Synthetic_FoldX_ΔΔG_ 100000_substitution_type_min			minimize antibody substitution type diversity			1,293 (Train + Val)	16	48,747, 20,314, 14,940, 5,999
Synthetic_FoldX_ΔΔG_ 100000_substitution_type_max			maximize antibody substitution type diversity			1,324 (Train + Val)	380	35,549, 12,464, 27,987, 14,000
Synthetic_FoldX_ΔΔG_ 100000_mutation_ distribution_min			minimize antibody mutation distribution diversity			1,321 (Train + Val)	380	0, 0, 90,000, 0
Synthetic_FoldX_ΔΔG_ 100000_mutation_ distribution_max			maximize antibody mutation distribution diversity			1,324 (Train + Val)	380	22,505, 22,498, 22,499, 22,498
Synthetic_FoldX_ΔΔG_ 100000_randomly_sampled		Train and validation datasets randomly sampled from Synthetic_FoldX_ΔΔG_ 942723, for which no complex overlaps with any in Synthetic_FoldX_ΔΔG_100000_ diversity_test_set				1,332 (Train + Val)	380	31,283, 14,800, 31,345, 12,572
Synthetic_FoldX_ΔΔG_ 100000_diversity_test_set		Test dataset for all train/val diversity datasets – consists of 10,000 mutations, for which no complex overlaps with any in the train and validation sets			Test: 10,000	139 (Test)	380	3,468, 3,377, 1,763, 1,392
Synthetic_FlexddG_ΔΔG_ 20829	Synthetic (Flex ddG)	Synthetic single-point mutation ΔΔG data generated using Rosetta Flex ddG		None (Random), 100%, 90%, 70%	20,829	1,302	380	6,799, 3,389, 7,800, 2,841
Synthetic_FlexddG_ΔΔG_580		Train + validation datasets of varying sizes randomly sampled from the respective Synthetic_FlexddG_ΔΔG_20829 datasets		90%	580	461	272	180, 93, 227, 80
Synthetic_FlexddG_ΔΔG_900					900	623	319	296, 147, 338, 119
Synthetic_FlexddG_ΔΔG_4500					4,500	1,155	375	1,504, 701, 1,711, 584
Synthetic_FlexddG_ΔΔG_9000					9,000	1,169	380	3003, 1419, 3392, 1186
Synthetic_FlexddG_ΔΔG_2083		Test dataset to which models trained on the train + validation datasets of varying sizes were applied			2,127	133	356	593, 406, 768, 360

Supplementary Table 2: The performance of ΔΔG prediction methods trained on experimental antibody-antigen ΔΔG datasets with a 90% CDR sequence identity train-validation-test cutoff. This analysis includes models which could be retrained with the data splits from this study. The models were trained and tested on the 'Experimental_ΔΔG_645 − Reverse Mutations + Non-Binders' (AB-Bind) and 'Experimental_ΔΔG_608' (SKEMPI) datasets with 10-fold cross-validation. For the Experimental_ΔΔG_645 dataset, the correlations are also included for the test dataset excluding the non-binder mutations. For more information, see Methods ('Comparison against protein-protein interaction ΔΔG prediction methods').

Test Pearson's Correlation (10-Fold Cross-Validation)

	**Experimental_ΔΔG_645**		**Experimental_ΔΔG_608**
	**Including non-binders**	**Excluding non-binders**	
**DGCddG**	0.26	0.32	0.45
**RDE-PPI**	0.22	0.35	0.48
**Graphinity**	-0.02	0.19	0.38

Supplementary Table 3: Synthetic ΔΔG dataset generation runtimes. Times are given in HH:MM:SS and the ratio represents $\frac{\text{Flex ddG Runtime}}{\text{FoldX Runtime}}$.

	**FoldX**	**Flex ddG**	**Ratio**
**Mean**	00:00:17	10:20:35	2146.30
**Median**	00:00:14	02:17:14	586.07

Supplementary Table 4: The performance of different models on the Synthetic_FoldX_ΔΔG_942723 dataset (one fold, held-out test set; 90% length-matched CDR sequence identity cutoff). The same train, validation, and test dataset was used for each model.

**Model Input**	**Model**	**Test Pearson's Correlation**
One-hot encoded interface residues	FLAML	0.27
	CNN	0.16
ESM2 embedding of mutated position in WT sequence	FLAML	0.44
Embeddings from normalizing flows	RDE-PPI	0.64
Graph of mutation neighborhood	Equiformer	0.89
	Graphinity (EGNN)	0.87

Supplementary Table 5: Pharmacophore counts for each amino acid, as used in the tree-based model featurization.

AA	Neutral	H-bond Donor	H-bond Acceptor	Hydro-phobic	Aromatic	Positive	Negative	Sulphur
A	2	1	1	1	0	0	0	0
C	2	1	1	1	0	0	0	1
D	3	1	3	1	0	0	2	0
E	3	1	3	2	0	0	2	0
F	2	1	1	7	6	0	0	0
G	2	1	1	0	0	0	0	0
H	2	3	3	6	5	2	0	0
I	2	1	1	4	0	0	0	0
K	2	2	1	4	0	1	0	0
L	2	1	1	4	0	0	0	0
M	2	1	1	4	0	0	0	0
N	3	2	2	1	0	0	0	0
P	1	0	1	5	0	0	0	0
Q	3	2	2	2	0	0	0	0
R	3	4	1	3	0	3	0	0
S	2	2	2	1	0	0	0	0
T	2	2	2	2	0	0	0	0
V	2	1	1	3	0	0	0	0
W	2	2	1	10	9	0	0	0
Y	2	2	2	7	6	0	0	0

Supplementary Table 6: Structural changes resulting from modeling of mutations using FoldX and Flex ddG. The RMSD calculation was performed for atoms within 10 Å of the C_α atom of the mutated residue. This calculation was completed for the 20,829 mutants which were modeled with both FoldX and Flex ddG using the Pymol align function.

**WT**	**Mutant**	**RMSD (Mean)**	**RMSD (Median)**
Solved	FoldX BuildModel	0.003	0.000
FoldX Repaired	FoldX BuildModel	0.000	0.000
Solved	Flex ddG Modelled	0.329	0.302
Flex ddG Modelled	Flex ddG Modelled	0.046	0.035

Supplementary Results

SKEMPI 2.0 benchmark dataset

Although widely used, the AB-Bind dataset suffers from several limitations, including that five of the included complexes do not contain an antibody, despite the dataset being described as an "antibody binding mutational database" [2] (more details in Methods, 'Experimental $\Delta\Delta$G data preparation'). We therefore propose an experimental antibody-antigen single-point mutation $\Delta\Delta$G dataset (Experimental_$\Delta\Delta$G_608, Supplementary Table 1, Supplementary Figure 1B), consisting of 608 mutations filtered from the SKEMPI 2.0 database [3], for model benchmarking. Although this dataset has fewer single-point mutations, these mutations come from a larger number of complexes (44). The performance of Graphinity on this dataset is similar to that for the Experimental_$\Delta\Delta$G_645 dataset (Supplementary Figure 3A): model correlation is high when the data is split randomly and with reverse mutations, but once again is not robust to train-test CDR sequence identity cutoffs.

On this more rigorous dataset, we also investigated the robustness of models trained on featurized inputs. We built a tree-based model from features (more details in Methods, 'Tree-based model trained on featurized structures') derived from the WT and mutant complex structures, similar to the method employed by the mCSM-based models [4, 5]. This different model architecture gave similar correlations and also suffered from overtraining (Supplementary Figure 3B).

Model performance on evolutionarily grounded mutations

A recent study found that FoldX accuracy was higher for mutations that are observed naturally, with an 11% decrease in incorrectly predicting a mutation to be stabilizing [6].

The performance of Graphinity was stable on a test dataset limited to such 'evolutionarily grounded' mutations from human and mouse sequences (see Methods, 'Evolutionarily grounded mutations'), with a Pearson's correlation of 0.91. Conversely, Graphinity also performed well (Pearson's correlation = 0.88) on non-evolutionarily grounded mutations from human and mouse sequences.

Affinity-based train-validation-test split

We trained Graphinity on the lowest 80% of $\Delta\Delta$G values in the Synthetic_FoldX_$\Delta\Delta$G_942723 dataset, validated on the next 10%, and tested on the top 10%. The model achieved some separation (Pearson's correlation = 0.52), but only correctly identified 21% of the mutations as improving affinity (Supplementary Figure 5).

Synthetic Flex ddG dataset

We generated synthetic antibody-antigen $\Delta\Delta$G datasets using FoldX [3] and Rosetta Flex ddG [7]. The modeled structures produced by Flex ddG exhibited greater structural changes from (higher RMSD with) the initial solved structures as compared to those output by FoldX. While there is some difference observed between the WT and mutant structures around the mutated site (not seen in the FoldX structures), a substantial portion of these structural changes appear to result from the relaxing of the overall structure and are found in the WT model as well (Supplementary Table 6).

On the dataset of 20,829 Flex ddG mutations (Synthetic_FlexddG_$\Delta\Delta$G_20829, Supplementary Table 1, Supplementary Figure 1D), Graphinity achieved a moderate Pearson's correlation of 0.70 with a 90% CDR sequence identity cutoff (Supplementary Figure 7; model training for 500 epochs). The model was stronger on a FoldX dataset comprised of the equivalent 20,829 mutations (Pearson's correlation = 0.88; model training for 500 epochs), which may arise from a more complex energy function and/or conformational sampling (including of the backbone) in Flex ddG. Flex ddG is typically implemented by averaging across multiple (default 35) structures, and individual structure models may also be noisy. For the large synthetic dataset generated in this study, computational requirements restricted the number of models we could generate to one per mutation. As a result, there may be a higher level of noise in the Flex ddG values, contributing to the lower predictive accuracy.

Model performance on the FoldX values for the subset of the synthetic mutations generated with Flex ddG was higher than on the datasets of equivalent size sampled from the full FoldX dataset (Figure 3A, Supplementary Figure 7). This may result from the longer training times (500 rather than 10 epochs, feasible due to the smaller dataset size), as well as the reduced number of antibody-antigen complexes in the Flex ddG dataset (1302 rather than 1471 complexes, due to Flex ddG failing for some PDBs) and thus an easier prediction task.

Performance by amino acid substitution

We trained models on synthetic FoldX datasets limited to each amino acid substitution type (for example, Arg to Lys) to explore whether Graphinity could learn the effect of a substitution better when trained only on data for this substitution. However, model performance for a specific substitution decreased as compared to the model trained on the full dataset (Supplementary Figure 9). Performance could be rescued, reaching or exceeding that of the model trained on the full dataset, by initializing with weights from the full model (Supplementary Figure 9).

References

[1] Mason, D. M. *et al.* Optimization of therapeutic antibodies by predicting antigen specificity from antibody sequence via deep learning. *Nature Biomedical Engineering* **5**, 600–612 (2021).

[2] Sirin, S., Apgar, J. R., Bennett, E. M. & Keating, A. E. AB-Bind: Antibody binding mutational database for computational affinity predictions. *Protein Science* **25**, 393–409 (2016).

[3] Schymkowitz, J. *et al.* The FoldX web server: An online force field. *Nucleic Acids Research* **33**, 382–388 (2005).

[4] Pires, D. E. & Ascher, D. B. mCSM-AB: a web server for predicting antibody–antigen affinity changes upon mutation with graph-based signatures. *Nucleic Acids Research* **44**, W469–W473 (2016).

[5] Myung, Y., Rodrigues, C. H., Ascher, D. B. & Pires, D. E. mCSM-AB2: Guiding rational antibody design using graph-based signatures. *Bioinformatics* **36**, 1453–1459 (2020).

[6] Rosace, A. *et al.* Automated optimisation of solubility and conformational stability of antibodies and proteins. *Nature Communications* **14**, 1937 (2023).

[7] Barlow, K. A. *et al.* Flex ddG: Rosetta Ensemble-Based Estimation of Changes in Protein-Protein Binding Affinity upon Mutation. *Journal of Physical Chemistry B* **122**, 5389–5399 (2018).
