# Supplementary material for: Investigating the volume and diversity of data needed for generalizable antibody–antigen ΔΔG prediction
Source: Nat Comput Sci. 2025 Jul 8;5(8):635–47. doi: 10.1038/s43588-025-00823-8 (PMC12374841; doi:10.1038/s43588-025-00823-8)
Supplement: Supplementary file 2 — Reporting Summary [file 43588_2025_823_MOESM2_ESM.pdf]

nature portfolio

Corresponding author(s): Alissa M. Hummer, Charlotte M. Deane

Last updated by author(s): 04/17/2025

Reporting Summary

Nature Portfolio wishes to improve the reproducibility of the work that we publish. This form provides structure for consistency and transparency in reporting. For further information on Nature Portfolio policies, see our Editorial Policies and the Editorial Policy Checklist.

Statistics

For all statistical analyses, confirm that the following items are present in the figure legend, table legend, main text, or Methods section.

n/a	Confirmed	
☐	☒	The exact sample size (*n*) for each experimental group/condition, given as a discrete number and unit of measurement
☒	☐	A statement on whether measurements were taken from distinct samples or whether the same sample was measured repeatedly
☒	☐	The statistical test(s) used AND whether they are one- or two-sided<br>*Only common tests should be described solely by name; describe more complex techniques in the Methods section.*
☒	☐	A description of all covariates tested
☒	☐	A description of any assumptions or corrections, such as tests of normality and adjustment for multiple comparisons
☒	☐	A full description of the statistical parameters including central tendency (e.g. means) or other basic estimates (e.g. regression coefficient) AND variation (e.g. standard deviation) or associated estimates of uncertainty (e.g. confidence intervals)
☒	☐	For null hypothesis testing, the test statistic (e.g. *F*, *t*, *r*) with confidence intervals, effect sizes, degrees of freedom and *P* value noted<br>*Give P values as exact values whenever suitable.*
☒	☐	For Bayesian analysis, information on the choice of priors and Markov chain Monte Carlo settings
☒	☐	For hierarchical and complex designs, identification of the appropriate level for tests and full reporting of outcomes
☐	☒	Estimates of effect sizes (e.g. Cohen's *d*, Pearson's *r*), indicating how they were calculated

Our web collection on statistics for biologists contains articles on many of the points above.

Software and code

Policy information about availability of computer code

Data collection	We modeled mutations and predicted the corresponding change in binding affinity values for antibody-antigen complexes from the SAbDab database (accession date 19 May 2022) using FoldX (version 5) and Rosetta Flex ddG (Rosetta version 2020.08+release.cb1caba).
Data analysis	We built the EGNN deep learning models in Python v3.7.10 using PyTorch v1.8.0 and PyTorch Geometric v1.6.3. The code is available at https://github.com/oxpig/Graphinity.<br>We visualized the data using Python v3.9.1, matplotlib v3.9.2, seaborn v0.13.2, PyMOL v2.3.0, and PowerPoint v16.95.4. Colors were selected in part using ColorBrewer 2.0 (https://colorbrewer2.org).

For manuscripts utilizing custom algorithms or software that are central to the research but not yet described in published literature, software must be made available to editors and reviewers. We strongly encourage code deposition in a community repository (e.g. GitHub). See the Nature Portfolio guidelines for submitting code & software for further information.

Data

Policy information about availability of data

All manuscripts must include a data availability statement. This statement should provide the following information, where applicable:

- Accession codes, unique identifiers, or web links for publicly available datasets
- A description of any restrictions on data availability
- For clinical datasets or third party data, please ensure that the statement adheres to our policy

The synthetic ΔΔG datasets and links to download the corresponding PDBs can be found at https://github.com/oxpig/Graphinity.

The AB-Bind database is available at https://github.com/sarahsirin/AB-Bind-Database. The SKEMPI v2.0 database is available at https://life.bsc.es/pid/skempi2.

Source data for Figures 2-4 is available with this manuscript and at https://github.com/oxpig/Graphinity.

Human research participants

Policy information about studies involving human research participants and Sex and Gender in Research.

Reporting on sex and gender	NA – no studies involving human participants were conducted for this work.
Population characteristics	NA – no studies involving human participants were conducted for this work.
Recruitment	NA – no studies involving human participants were conducted for this work.
Ethics oversight	NA – no studies involving human participants were conducted for this work.

Note that full information on the approval of the study protocol must also be provided in the manuscript.

Field-specific reporting

Please select the one below that is the best fit for your research. If you are not sure, read the appropriate sections before making your selection.

☒ Life sciences ☐ Behavioural & social sciences ☐ Ecological, evolutionary & environmental sciences

For a reference copy of the document with all sections, see nature.com/documents/nr-reporting-summary-flat.pdf

Life sciences study design

All studies must disclose on these points even when the disclosure is negative.

Sample size	We created synthetic change in binding affinity datasets using PDBs from SAbDab. The final datasets consisted of 942,723 FoldX mutations from 1471 antibody-antigen complexes and 20,829 Flex ddG mutations from 1302 antibody-antigen complexes.<br><br>The synthetic FoldX dataset size was determined as the number of mutations that could be generated through exhaustive mutagenesis of interface positions from a clustered dataset (90% length-matched CDR sequence identity cutoff) of solved antibody-antigen complex structures. The synthetic Flex ddG dataset was subsampled from the synthetic FoldX dataset at ca. 16 mutations per antibody-antigen complex. The Flex ddG dataset size was restricted by computational requirements.<br><br>The sufficiency of these datasets was analyzed through developing models on subsampled datasets. Performance plateaued as the full dataset size was reached.<br><br>Analyses were also conducted on existing experimental databases, AB-Bind and SKEMPI 2.0.
Data exclusions	We clustered the SAbDab PDBs based on 90% length-matched CDR sequence identity and carried forward one PDB from each cluster for interface mutagenesis. We excluded mutations where the WT amino acid was 'X', the chain identifier was a number, the antibody and antigen were > 4 A apart and the FoldX Interaction Energy calculation or Rosetta Flex ddG mutation modeling failed.
Replication	The FoldX software is deterministic.
Randomization	This study is centered on the development and validation of models for predicting change in antibody-antigen binding affinity. The quantitative data is formatted as the difference in binding affinity between the wild-type and mutant complexes (ΔΔG) and does not test for a causal relationship between groups. As such, randomization was not applicable to this study.
Blinding	This study is centered on the development and validation of models for predicting change in antibody-antigen binding affinity. The quantitative data is formatted as the difference in binding affinity between the wild-type and mutant complexes (ΔΔG) and does not test for a

March 2021

causal relationship between groups. As such, blinding was not applicable to this study.

Reporting for specific materials, systems and methods

We require information from authors about some types of materials, experimental systems and methods used in many studies. Here, indicate whether each material, system or method listed is relevant to your study. If you are not sure if a list item applies to your research, read the appropriate section before selecting a response.

Materials & experimental systems

n/a	Involved in the study
☒	☐ Antibodies
☒	☐ Eukaryotic cell lines
☒	☐ Palaeontology and archaeology
☒	☐ Animals and other organisms
☒	☐ Clinical data
☒	☐ Dual use research of concern

Methods

n/a	Involved in the study
☒	☐ ChIP-seq
☒	☐ Flow cytometry
☒	☐ MRI-based neuroimaging
